# Ecological Risk and Early Warning of PCBs in Central Jilin Province’s Black Soil Zone, China

**DOI:** 10.3390/toxics13040249

**Published:** 2025-03-27

**Authors:** Jinying Li, Yanan Chen, Dianqi Pan, Jiquan Zhang, Yichen Zhang, Pengju Song, Wanying Shi

**Affiliations:** 1 Changchun Institute of Technology, College of Jilin Emergency Management, Changchun 130012, China; 20231311206@stu.ccit.edu.cn (J.L.); zhangjq022@nenu.edu.cn (J.Z.); zhangyc@ccit.edu.cn (Y.Z.); 20231091221@stu.ccit.edu.cn (P.S.); 20231311212@stu.ccit.edu.cn (W.S.); 2 Institute of Natural Disaster Research, School of Environment, Northeast Normal University, Changchun 130024, China

**Keywords:** black soil, polychlorinated biphenyls, source analysis, risk assessment, early warning

## Abstract

To investigate the levels of polychlorinated biphenyls (PCBs) in the black soils of Northeast China, we collected 59 surface soil samples from the central black soil region of Jilin Province. We analyzed the concentrations and sources of seven PCBs in the black soil, assessed the ecological risks associated with PCB contamination, and provided a risk assessment for PCBs in this soil type. The mean concentrations of the seven PCBs (PCB28, PCB52, PCB101, PCB118, PCB138, PCB153, and PCB180) were as follows: 1.61 μg/kg, 10.61 μg/kg, 0.37 μg/kg, 4.11 μg/kg, 0.70 μg/kg, 1.07 μg/kg, and 2.09 μg/kg, respectively. Principal component analysis revealed that PCB contamination in black soil is mainly attributed to automobile exhaust emissions during transportation, waste incineration processes, and insulation materials from electronic and electrical equipment. PCB28 and PCB52 are the primary causes of PCB danger, according to the findings of the ecological risk assessment, with Liaoyuan City having the highest risk. By applying contemporary industrial economic theory to analyze the annual accumulation of contaminants, we forecasted future PCB concentrations in black soil and issued a risk warning for these seven PCBs. Our results indicate that under the three scenarios considered, the presence of these seven PCBs in black soil does not pose a significant risk. However, given that our study examined only seven PCBs, the actual environmental risk may be underestimated.

## 1. Introduction

Polychlorinated biphenyls (PCBs) are designated as synthetic aromatic molecules composed of carbon, hydrogen, and chlorine [1]. Owing to their high thermal stability and excellent electrical insulation properties, PCBs have been widely utilized in applications such as additives in pesticides, plasticizers, and insulation materials [2,3,4]. As persistent organic pollutants, PCBs can present health risks to humans, even at low concentrations [5,6]. Existing studies have demonstrated that exposure to polychlorinated biphenyls (PCBs) can lead to tumors, immune deficiencies, endocrine disorders, gastrointestinal diseases, and liver diseases [7,8]. While many countries have completely banned the production and use of PCBs, legacy contamination remains a significant issue. The rapid advancement of industry has led to an increase in waste and pollutants being converted into PCBs and related compounds, which subsequently enter the soil via diverse pathways. Given the propensity of organic pollutants to be adsorbed by soil particles [9,10], researchers have identified soil as a crucial medium for the detection of PCBs, serving as a significant sink for these contaminants [11].

Several researchers have explored the concentrations, sources, ecological hazards, and health risks associated with PCBs across diverse environmental matrices, such as water bodies [12,13,14,15], soil [16,17], and the atmosphere [18,19,20]. PCB concentrations were determined following EU standard methods [21], and their sources were analyzed using principal component analysis [6,13]. The ecological hazard level was assessed using the risk quotient (RQ) [6] and the contamination severity index (CSI) [22], whereas health risks were evaluated based on the health quotient (HQ), as outlined by the U.S. Environmental Protection Agency [23]. The notable variations in PCB concentrations observed across different regions can likely be attributed to differences in economic development, population density, and the specific locations of sampling sites.

China’s agricultural development predominantly focuses on the Northeast region, which is renowned for its fertile black soil. This black soil plays a crucial role in China’s agricultural productivity. However, in recent years, rapid industrial growth coupled with the extensive use of pesticides in agricultural practices has led to the detection of PCBs in this fertile soil. Unlike many other organic pollutants, PCBs can present health risks to humans, even at very low concentrations. Furthermore, PCBs can enter the human body via the consumption of crops and other agricultural produce, potentially causing health problems. Consequently, assessing the risk posed by PCB residues in the black soil of central Jilin Province is essential. Previous studies have predicted the future risks of pollutants using methods such as mass balance models [24] and machine learning [25,26]. However, the performance of these methods varies significantly and is limited by data availability. In this study, a dynamic relationship model between economic activity and ecological risk is established by using GDP as a proxy variable for pollution source activity intensity (AI). Additionally, the economic activity index (GDP), pollution emission intensity (EI), and ecological risk index (CSI) are integrated into a dynamic evaluation framework. The study examines the trend of PCB ecological risk under different economic growth scenarios (GDP 6% and 3% growth rates), providing a reference for mitigating the risks caused by PCB residues.

In contrast to previous studies that have focused on agricultural soils, this research centers on the black soil of central Jilin Province, with an emphasis on analyzing PCB residues and forecasting future PCB trends in this region. The primary objectives of this study are, therefore, (1) to investigate the relationship between PCB concentrations and soil properties such as pH and organic matter content, (2) to conduct source analysis of PCBs, (3) to assess the ecological risks associated with PCB contamination, and (4) to perform a risk warning evaluation for PCBs.

## 2. Materials and Methods

### 2.1. Study Area and Sample Collection

**Figure 1:**
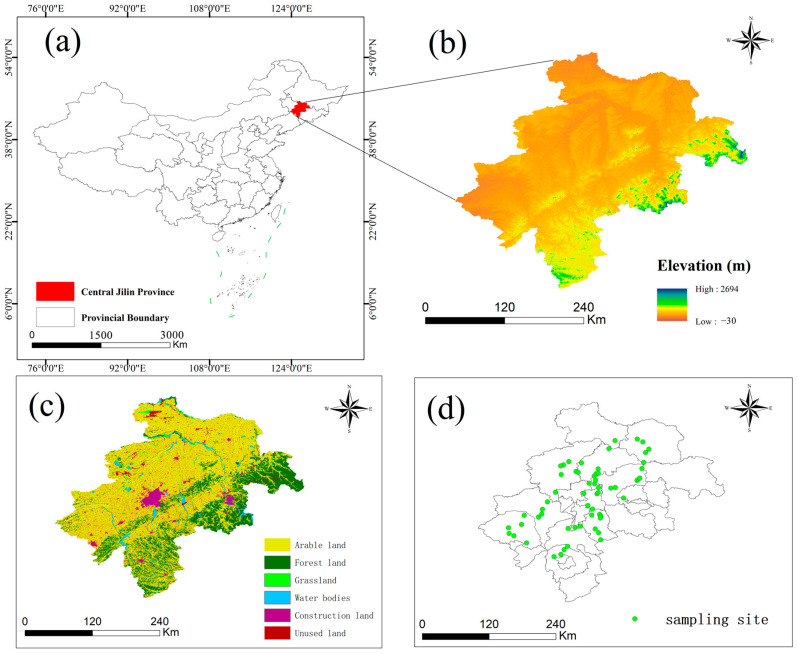
Map of the study area and sampling sites (**a**) Central Jilin Province, China (**b**) Elevation (**c**) Land use type (**d**) Sampling point.

Located in Northeast China, Jilin Province has a temperate continental monsoon climate, distinguished by its four distinct seasons and concurrent rainfall and warmth during the growing season [27]. The region receives an average annual precipitation of approximately 567 mm, with average temperatures ranging from 3 to 5 degrees Celsius. The predominant soil types for agriculture in this area are black calcareous soil and black soil. Meanwhile, the key industries supporting the regional economy include automobile manufacturing, petrochemicals, and the processing of agricultural products. In the central Jilin Province’s black soil region, we collected 59 surface soil samples (Figure 1). Surface soil samples (0–20 cm depth) were collected from farmland in the black soil region. At each site, a composite soil sample was composed of five individual soil samples, which were taken from a square plot with a side length of 1 km (four samples from the corners and one from the center, each weighing 1 kg). Fresh soil samples were transported to the laboratory in high-density polyethylene (HPPE) bags, with sampling sites selected randomly. Samples were prepared by removing larger stones and debris, passing them through a 100-mesh sieve, and storing them at low temperatures prior to analysis.

### 2.2. Sample Analysis

In this study, seven PCBs (PCB28, PCB52, PCB101, PCB118, PCB138, PCB153, and PCB180) were detected in the black soil region of central Jilin Province. Throughout the pre-treatment and analysis of soil samples, we strictly adhered to the protocols set by the Ministry of Ecology and Environment [28].

Weigh 20 g (to the nearest 0.01 g) of a 1 mm sieve soil sample into a 250 mL conical flask and add an appropriate amount of anhydrous sodium sulfate for dehydration. Add 40.0 μg of substitute, dissolved in an acetone–hexane solvent mixture (1:1, *v*/*v*). Add 100 mL of the acetone–hexane solvent mixture to the conical flask. Perform ultrasonic extraction for 20 min, filter the extract, and collect it in a concentration apparatus. Wash the container with the same solvent mixture as above. Reduce the extract to approximately 2 mL using a rotary evaporator, then add 10 mL of hexane and concentrate the mixture to 2 mL before proceeding with purification. First, purify the darker-colored concentrate using sulfuric acid (which removes most oxygenated compounds and some organochlorine pesticides). Transfer the concentrate to a 150 mL separatory funnel, add 10 mL of sulfuric acid, and gently shake it for 1 min. Allow the layers to separate and discard the sulfuric acid layer. The magnesium silicate solid-phase extraction column was washed with 8 mL of hexane to keep the surface of adsorbent in the column moistened, and the concentrate was transferred to the extraction column for 1 min, and then the effluent was discarded. Add 2 mL of the acetone–hexane solvent mixture (acetone:hexane/v:v/1:9), let it sit for 1 min, collect the cleanup solution, and wash the extraction column with the above solvent until the volume of the collected cleanup solution reaches 10 mL. Concentrate the cleanup solution with a rotary evaporator, add the internal standard, and condense it to 1.0 mL. Transfer the final solution to a sample vial for analysis.

A gas chromatography–mass spectrometer (GC-MS, Clarus 680/600 T, PerkinElmer Inc., Waltham, MA, USA) was used to analyze the liquids that were to be analyzed. The gas chromatographic conditions were as follows: an injection port temperature of 250 °C, an initial temperature of 100 °C held for 2 min, a temperature increase of 15 °C/min up to 220 °C, a hold at that temperature for 5 min, followed by a temperature drop to 260 °C over 20 min. The temperature of the column was 100 °C for 2 min, 220 °C for 5 min at a rate of 15 °C/min, and finally 260 °C for 20 min at the same rate. Soil pH was measured with a pH electrode (Model PB-10, Sartorius, Gottingen, Germany) in a 1:2.5 (soil/distilled water) extract, and soil organic matter (OM) content was analyzed based on residual titration.

### 2.3. Quality Assurance and Quality Control

Before each usage, the glassware was washed with a solution of hexane and acetone in a ratio of 1:1, and then subjected to ultrasonic cleaning and drying at 400 °C. As part of the sample analysis, both blank and parallel sample tests were conducted. One blank sample was processed for every five samples, and it was found that the blank samples did not contain any target chemicals. With limits of detection (LODs) ranging from 0.01 to 0.21 ng/g, the PCB recoveries ranged from 80.9% to 106.4%. The relative standard deviation (RSD) was less than 5%.

### 2.4. Ecological Risk Assessment

The ecological risk of soil pollutants was assessed using the soil contamination severity index (CSI) [22,29,30]. In order to determine the severity of an impact on a biological community, this index uses the effect range low (ERL) and effect range medium (ERM) values [31]. Principal component analysis was used to obtain W_i_ for each PCB. ERM designates a concentration at which negative consequences are expected to occur frequently, whereas ERL designates a concentration below which negative effects are expected to occur infrequently. The following is the calculating formula:

(1) $$CSI=\sum_{i=1}^{n}W_{i}\times [{\left(\frac{C_{i}}{{ERL}_{i}}\right)}^{\frac{1}{2}}+{\left(\frac{C_{i}}{{ERM}_{i}}\right)}^{2}]$$

In the formula, $W_{i}$ represents the weight of each PCB, $C_{i}$ denotes the concentration of each PCB, and ERL and ERM refer to the effect range low and effect range medium, respectively. Uncontaminated (CSI < 0.5), very low severity (0.5 ≤ CSI < 1), low severity (1 ≤ CSI < 1.5), low to moderate severity (1.5≤ CSI <2), moderate severity (2≤ CSI < 2.5), moderate to high severity (2.5≤ CSI <3), high severity (3≤ CSI <4), very high severity (4≤ CSI <5), and ultra-high severity (5≤ CSI) are the nine pollution levels classified according to the CSI values. Factor analysis and principal component analysis (PCA), which solely take anthropogenic variables into account when determining the weight values, are used to determine the weights of each PCB. The following formula is used to determine each PCB’s weight:

(2) $$W_{i}=\frac{\left({Loading\;value}_{i}\times eigen\;value\right)}{\sum_{i}^{n}\left({Loading\;value}_{i}\times eigen\;value\right)}$$

### 2.5. Temporal Dynamic Trends of PCBs in Soil

#### 2.5.1. Dynamic Source Release Modelling

Assuming that under natural attenuation conditions, pollutants diffuse from active pollution sources into the surrounding soil [32], analyzing this process requires examining the emission intensity (EI) of the pollution sources. The activity intensity (AI) of the polluting source multiplied by the time integral of the decay coefficient yields the environmental impact (EI) [33], with a positive correlation existing between EI and AI [34]. All of Jilin Province’s manufacturing operations are considered potential pollution hotspots in this analysis. An annual evaluation of advancements in the AI is carried out in order to study the dynamics of EI trends [35]. The AI is positively correlated with gross domestic product (GDP) [36]; therefore, the calculation process for EI is as follows:

(3) $${EI}_{m}\propto \sum_{2002}^{m}{AI}_{m}\times K^{m-2002}$$

In the equation, ${EI}_{m}$ and ${AI}_{m}$ represent the emission intensity (EI) and activity intensity (AI) of the pollution source in year m (where m = 2002, 2003, …, 2045). K denotes the natural degradation rate of the pollutant (with K = 0.95).

Aside from Changchun, the GDP of other cities in central Jilin Province did not show a significant growth trend. To highlight the temporal trend of pollutants, the AI data from 2002 to 2022 utilized the GDP of Changchun [37], and scenario simulations were conducted to predict AI values from 2023 to 2045. The scenario simulations included three scenarios: (a) fitting historical data to predict future values; (b) a 6% annual GDP growth rate starting in 2023; and (c) a 3% annual GDP growth rate starting in 2023.

#### 2.5.2. Time-Dynamic Trends in PCB Accumulation

The yearly contribution rate of pollution sources to soil pollutants can be calculated by observing the direct correlation between EI and this rate. The temporal distribution of pollutants can be predicted using the following formula:

(4) Pm,i=P¯2022,i×(∑2022mEImEI2022)×Km−2022

In the equation, $P_{m,i}$ represents the average concentration of the i-th pollutant in the soil of Changchun City in the year m.

### 2.6. Statistical Analysis

Using ArcGIS 10.7 (ESRI, Redlands, CA, USA) software, inverse distance weighting interpolation was applied to map the spatial distribution of PCBs in the soil. Principal component analysis was conducted using SPSS 17.0 (SPSS Inc., Chicago, IL, USA), and correlation plots were created using Origin 2024b (Origin Inc., Asheboro, NC, USA) to explore the relationships between PCBs, soil organic matter, and pH.

## 3. Results and Discussion

### 3.1. PCB Concentration

**Figure 2:**
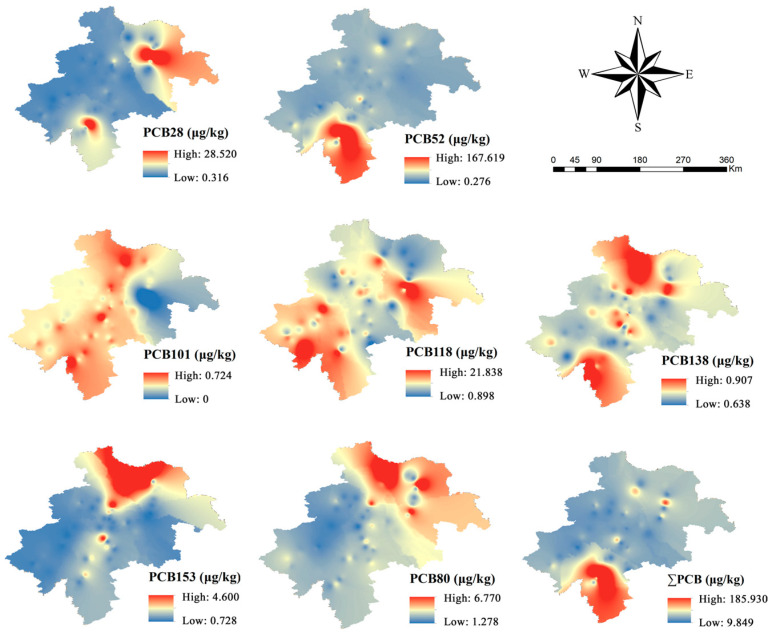
Geographical distribution of PCBs in the black soil area of central Jilin Province.

The concentration distribution of the seven PCBs in the agricultural soil of the black soil region in central Jilin Province is shown in Figure 2. The concentrations fluctuate with the sampling location, with the total concentration of the seven PCBs ranging from 9.849 to 185.930 μg/kg, and averaging 20.556 μg/kg. PCB28 and PCB52 were detected in Liaoyuan City, where PCB52 exhibited the highest concentration, at 167.619 μg/kg. This may be attributed to lower chlorinated PCBs tending to volatilize into the atmosphere and disperse through air currents [4]. Small amounts of PCB28 were also found in Dehui and Yushu Cities. The concentration of PCB101 ranged from 0 to 0.724 μg/kg, indicating its relatively low presence in samples from the black soil region. PCB118 was predominantly found in the Jiutai and Tiexi Districts, whereas PCB138, PCB153, and PCB180 showed higher concentrations in Fuyu and Yushu Cities.

The concentration range for lower chlorinated PCBs (PCB28 and PCB52) was 1.27 to 179.44 μg/kg, averaging 12.22 μg/kg, which accounts for 60% of the total PCB concentrations. Conversely, the total concentration of higher chlorinated PCBs (PCB101, PCB118, PCB138, PCB153, and PCB180) was 491.72 μg/kg, with an average of 8.33 μg/kg, constituting 40% of the total PCB concentrations. Due to their lower molecular weight and higher volatility, lower chlorinated PCBs can readily volatilize from pollution sources into the atmosphere and be transported over long distances. This volatility enables lower chlorinated PCBs to more easily reach areas distant from the pollution sources, whereas higher chlorinated PCBs, due to their lower volatility, tend to accumulate more in soils near the pollution sources [38].

The global concentrations of PCBs are summarized in Table 1. The average concentration of PCB28 (1.61 μg/kg) was lower than that of Dilovasi, Turkey (1.72 μg/kg), and Birgunj, Nepal (2.01 μg/kg), and higher than that of areas such as Hong Kong (1.30 μg/kg), Tripura, India (0.49 μg/kg), Nigeria (0.78 μg/kg), and Hawaii, USA (0.58 μg/kg). The average concentration of PCB 52 (10.62 μg/kg) was higher than that of the above-mentioned areas, and the average concentration of PCB 101 (0.37 μg/kg) was higher than the average concentration of PCB 101 (0.19 μg/kg) in Tripura, India (0.19 μg/kg), Nigeria (0.19 μg/kg), India (0.15 μg/kg), Thailand (0.49 μg/kg). kg), and Nigeria (0.33 μg/kg). The mean concentration of PCB118 (4.10 μg/kg) was significantly higher than that of the above-mentioned districts, and the mean concentration of PCB138 (0.70 μg/kg) was higher than that of Tripura, India (0.13 μg/kg), and Birgunj, Nepal (0.10 μg/kg). The mean concentration of PCB153 (1.07 μg/kg) was lower than that of Hawaii, USA (3.13 μg/kg), but higher than that of the other regions, and the mean concentration of PCB180 (2.09 μg/kg) was also higher than that of the above regions. Overall, the seven PCB concentrations in this study were high.

### 3.2. Correlation Analysis

Soil pH and organic matter content are presented in Table 2. The sample soil pH generally falls within the neutral to slightly alkaline or slightly acidic range, while the organic matter data indicate that soil fertility in the study area is not uniformly distributed.

**Figure 3:**
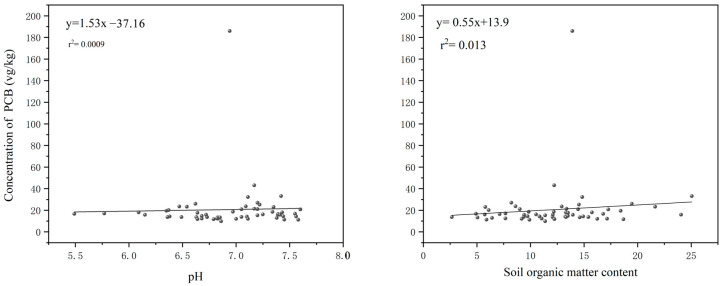
Correlation analysis of PCB concentration with pH and soil organic matter content.

To investigate the distribution variations of PCBs in soils across different regions, this study explored the relationships between soil organic matter content, pH, and PCB concentrations. The results suggest that a higher organic matter content enhances the binding of chemicals to soil particles [45], contributing to pollutant accumulation. A significant correlation was observed between PCB distribution and soil organic matter content [46,47]. In this study, no significant linear correlation was found between total PCB concentrations and soil pH (Figure 3), indicating that pH variations have a minimal impact on PCB levels. However, a significant positive correlation was observed between PCB concentrations and organic matter content. This is likely due to the high organic matter content in black soils, which enhances the binding of PCBs to soil particles.

### 3.3. PCB Source Analysis

In this study, SPSS 27 was utilized to conduct principal component analysis (PCA), a method widely employed to identify the sources of various organic contaminants [6,48,49]. To assess the suitability of the original data for PCA, the Kaiser–Meyer–Olkin (KMO) measure of sampling adequacy and Bartlett’s test of sphericity were conducted. The results indicated that the original data were appropriate for PCA, with a KMO value exceeding 0.5 and a significant Bartlett’s test result (*p* < 0.001).

A total of seven PCBs were identified in the farmland soils of the black soil region in central Jilin Province. A three-dimensional PCA analysis was performed on these seven congeners, yielding a cumulative variance contribution of 66.88% (Table 3 and Table 4). In the black soil region of central Jilin Province, PC1 comprised PCB101, PCB138, and PCB153; PC2 included PCB-28 and PCB52; and PC3 consisted of PCB118 and PCB180. The variance contribution rates for PC1, PC2, and PC3 were 27.83%, 21.13%, and 17.92%, respectively. IPC1 exhibited the highest loadings for PCB101, PCB138, and PCB153, collectively accounting for 10.38% of the total PCB concentration. Previous studies indicate that PCBs containing 5–6 chlorine atoms are commonly found in emissions from waste incineration and gasoline engine operations [50]. Therefore, it can be inferred that PC1 likely originates from PCBs generated by anthropogenic activities, such as vehicle exhaust and waste incineration.

PC2 exhibited the highest loading for PCB28, which accounted for 7.82% of the total PCB concentration. Transformer oils are known to contain high concentrations of PCB28, which suggests that the soil contamination observed may be primarily attributed to the insulation materials used in electrical and electronic equipment [39].

PC3 showed the highest loadings for PCB118 and PCB180, collectively accounting for 30.16% of the total PCB concentration. PCB-containing discarded electronic devices (such as capacitors and transformers) may release PCB 118 and PCB 180 into the soil through mishandling or burning during informal dismantling processes [51]. Given that major industries and large electronic waste processing facilities are concentrated in the central region [52], atmospheric deposition could serve as a significant source of these two congeners.

### 3.4. Ecological Risk Evaluation of PCBs in Soil

**Figure 4:**
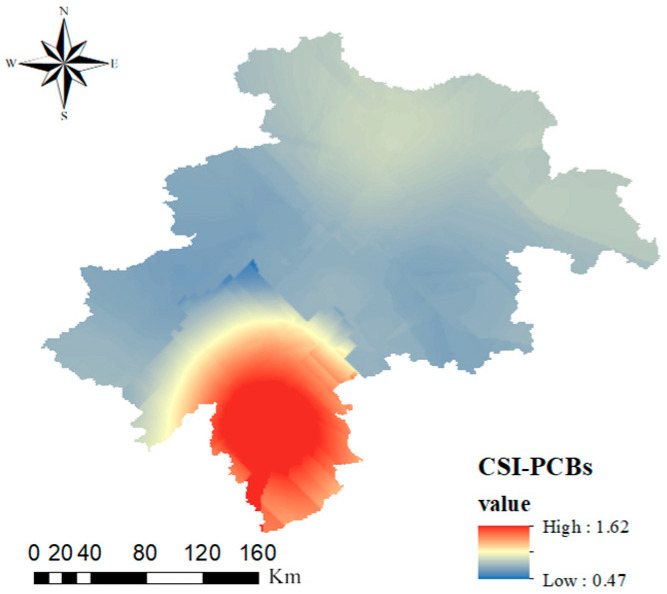
PCB contamination severity index (CSI) values distributed geographically in the central black soil zone of Jilin Province.

Figure 4 illustrates the geographic distribution of PCB-CSI values in the farmland soils of the black soil region in central Jilin Province. The highest CSI value was observed in Liaoyuan City, where only 1.7% of the samples fell into the “extremely high severity” category, with the majority classified as “very low severity”. Given the absence of ERL and ERM values for individual PCB congeners, this section focuses on the overall risk posed by total PCB concentrations. Based on the regional distribution maps presented in Section 3.1 and the analysis of farmland soils in the black soil region of central Jilin Province, PCB28 and PCB52 appear to be the primary contributors to the relatively high PCB-related risks. These congeners are commonly used as insulation fluids in capacitors and transformer oils. Once released into the soil, they can be readily transported by surface runoff [53]. Furthermore, highly chlorinated PCBs (PCB101, PCB118, PCB138, PCB153, and PCB180) can undergo dechlorination in the natural environment, potentially transforming into lower chlorinated congeners such as PCB28 and PCB52 [54].

### 3.5. PCB Risk Warning

**Figure 5:**
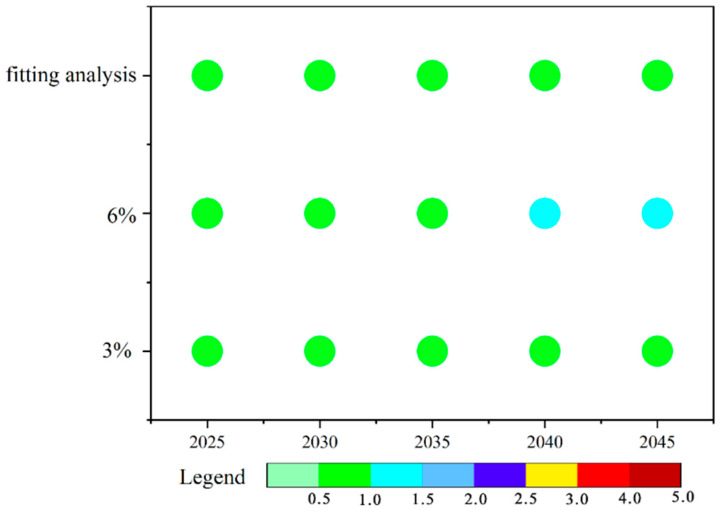
The predicted soil PCB contamination severity index (CSI), according to fitting studies and predictions; with a 6% annual GDP growth rate; and with a 3% annual GDP growth rate.

The projected future ecological risks of PCBs, based on annual average PCB concentrations, are depicted in Figure 5. To better illustrate these outcomes, three distinct scenarios were modeled, beginning with the year 2023. Under a scenario with an annual GDP growth rate of 6%, soil risk is expected to rise by one level by 2040, though it will remain within the “low risk” category. In the remaining two scenarios, the ecological risk levels are forecasted to remain stable and categorized as “low risk”. Despite the potential for increased industrial activity and associated PCB emissions under a 6% GDP growth scenario, we anticipate that stringent environmental protection policies and technological advances could mitigate any substantial increases in soil PCB concentrations, thereby maintaining low risk levels.

Since 1980, China has progressively phased out the use of electrical equipment and industrial products containing PCBs, alongside implementing a comprehensive suite of laws and regulations, such as the Soil Pollution Prevention and Control Law. This law outlines prevention and control measures for soil pollutants, including PCBs, delineates the responsibilities of both government entities and enterprises in soil pollution prevention, and establishes clear remediation standards. Additionally, a nationwide environmental monitoring network has been established to systematically track PCB levels in soil, water, and air. Monitoring data are utilized to evaluate pollution levels and inform targeted preventive actions. Furthermore, a series of pollution prevention measures and actions proposed by the government in the 2022 Jilin Environmental Bulletin [55], along with a 30% year-on-year decrease in illegal discharge cases, demonstrate that these efforts have significantly reduced the environmental risks posed by pollutants. Consequently, across all three scenarios, it is unlikely that PCB concentrations in soil will present significant ecological risks.

### 3.6. Uncertainty Analysis

This study’s prediction of soil risk may have certain limitations. These limitations stem from two primary factors: First, the GDP of Changchun was used as a proxy for the entire central region of Jilin Province, potentially leading to an overestimation of the actual economic activity and associated pollution levels. Second, the relationship between GDP growth and annual pollution levels is subject to change due to uncertainties in future economic development. This variability could result in either an overestimation or underestimation of the predicted soil ecological risk values compared to actual conditions. Despite these uncertainties, the overall findings suggest that there remains no significant ecological risk posed by PCBs in the soil.

## 4. Conclusions

The agricultural soils in the black soil region of central Jilin Province exhibit relatively low levels of polychlorinated biphenyl (PCB) contamination. Specifically, higher concentrations of PCB101, PCB138, PCB153, and PCB180 were observed in Fuyu City, while PCB28 was predominantly distributed in Yushu City. PCB118 showed elevated levels in Siping City and Jiutai District, and Liaoyuan City recorded the highest average concentration of PCB52. Overall, significant regional concentration variations were evident. Correlation analysis indicated no clear relationship between PCB concentrations and soil pH, but a positive correlation was identified with soil organic matter (SOM) content. The inherently high organic matter content of black soil enhances the adsorption of PCBs onto soil particles. The primary sources of PCBs in the study area include transportation, waste incineration, and atmospheric deposition. Human activities significantly influence the input levels of these contaminants. However, only 1.7% of the samples fall into the “extremely high severity” category; the spatial distribution of high-risk sites correlates strongly with historical industrial hotspots, suggesting localized contamination legacies. The early warning assessment further indicates that no significant ecological risks are expected to occur in central Jilin Province in the next 20 years under all three projection scenarios. This preliminary optimistic trend may stem from recent centralized governance efforts in high-priority zones, yet it should not overshadow the need for targeted interventions in lagging regions to address persistent disparities. This optimistic outlook is largely attributed to the ongoing efforts and effective measures implemented for soil pollution prevention and control in the study area.

## Figures and Tables

**Table 1:** Concentrations of PCBs in soil around the world (μg/kg).

Location	Average Concentration (μg/kg)							Reference
	PCB28	PCB52	PCB101	PCB118	PCB138	PCB153	PCB180	
Hong Kong	1.30	1.47	0.56	0.20	1.14	0.16	0.26	[39]
Dilovasi, Turkey	1.72	0.76	0.83	1.03	1.17	0.89	0.60	[40]
Tripura, India	0.49	0.49	0.19	0.26	0.13	0.20	0.05	[41]
Nigeria	0.78	0.46	0.33	0.84	0.22	0.54	0.31	[42]
Birgunj, Nepal	2.01	0.36	0.19		0.10	0.56	0.52	[43]
Hawaii, USA	0.58	0.54	0.91	1.58	3.12	3.13	2.05	[44]

**Table 2:** Description of soil pH and organic matter content.

	Max	Min	Average	Standard Deviation
pH	7.6	5.49	6.9	0.47
organic matter (g/kg)	25.06	2.67	12.23	4.64

**Table 3:** Interpretation of the total variance of the main principal.

	Rotating Parties and Loads		
Ingredient	Characteristic Root	Variance Rate (%)	Cumulative Contribution Rate (%)
1	2.216	27.83	27.83
2	1.351	21.13	48.95
3	1.114	17.92	66.88

**Table 4:** Component matrix after rotation.

	Principal Component		
Homologues	PC1	PC2	PC3
PCB28	0.024	0.830	−0.023
PCB52	0.110	0.745	−0.099
PCB101	0.749	0.192	−0.390
PCB118	−0.267	0.005	0.809
PCB138	0.686	0.406	0.157
PCB153	0.781	−0.066	−0.019
PCB180	0.473	−0.168	0.643

## Data Availability

The data presented in this paper are available on request from the corresponding authors.
